# Denosumab for Male Hemodialysis Patients with Low Bone Mineral Density: A Case-Control Study

**DOI:** 10.1155/2017/6218129

**Published:** 2017-08-22

**Authors:** Hiroya Takami, Kazunori Washio, Hiromichi Gotoh

**Affiliations:** ^1^Saiyu Kawaguchi Clinic, Kawaguchi, Japan; ^2^Saitama Honoka Clinic, Saitama, Japan; ^3^Saiyu Souka Clinic, Souka, Japan

## Abstract

Denosumab increases bone mineral density (BMD) in patients not receiving hemodialysis therapy. However, limited data are available in the literature concerning the use of denosumab in hemodialysis patients. We treated male hemodialysis patients with low radius BMD with denosumab therapy for 1 year and evaluated its effect on radius BMD. Seventeen patients were treated with denosumab 60 mg every 6 months, and 20 patients were not treated with denosumab (control group). At seven days, the mean corrected calcium level decreased from 9.2 ± 0.5 mg to 8.5 ± 0.5 mg (*P* < 0.01), and mean serum phosphorus decreased from 5.0 ± 1.3 mg/dl to 4.2 ± 0.9 mg/dl (*P* < 0.01). At 1 month, the corrected calcium and serum phosphorus levels were 9.2 ± 0.9 mg/dl and 4.0 ± 1.1 mg/dl, respectively. At 1 year, BMD increased by 2.6%  ± 4.4% in the denosumab group and decreased by 4.5%  ± 7.7% in the control group (*P* < 0.001). In our observational study, denosumab therapy represents an effective treatment for male dialysis patients with low BMD.

## 1. Introduction

Low bone mass is a worldwide public health concern that results in increased risk of fractures.

Bone fractures are relatively common among hemodialysis patients and pose a significant health burden [1–3]. Some studies suggested that bone mineral density (BMD) was lower in patients with chronic kidney disease who had fractures [4, 5].

Denosumab is a monoclonal antibody against the receptor activator of nuclear factor-*κβ* ligand (RANKL), a cytokine that is essential for the formation, function, and survival of osteoclasts [6]. By binding RANKL, denosumab prevents the interaction of RANKL with RANK on osteoclasts and reversibly inhibits osteoclast-mediated bone resorption. Many effective antiosteoporotic drugs are available, but, generally, they are contraindicated in patients with chronic kidney disease because of their progressive accumulation.

Although denosumab increased BMD in women without renal failure [7], effects in hemodialysis male patients are not well known.

Unlike other antiosteoporotic drugs, denosumab is not contraindicated in advanced chronic kidney disease, as its pharmacokinetics does not differ from that in patients with normal kidney function [8, 9]. Use of bisphosphonates in advanced chronic kidney disease requires considerable caution, and adequate clinical investigations were not reported [10]. A case report [11] and noncontrolled studies [12–14] indicated that denosumab could be efficacious in hemodialysis patients.

For 1 year, we used denosumab to treat male hemodialysis patients with low BMD at Saiyuu Kawaguchi Clinic and evaluated its effects on the BMD at the distal third of the radius in comparison with those in a control group.

As a hemodialysis patient treated with denosumab was reported to have developed severe hypocalcemia [15, 16], we carefully evaluated serum calcium levels.

## 2. Materials and Methods

This was an observational retrospective case-control study. At Saiyuu Kawaguchi Clinic in Japan, approximately 160 male patients underwent maintenance hemodialysis. Each patient was continuously taken care of by one of the two physicians. Each patient was randomly assigned to one of the two physicians at the first visit to the clinic. One physician administered treatment with denosumab, and the other physician did not use denosumab. Male patients with low BMD (<70% of the young adult mean) at Saiyuu Kawaguchi Clinic were eligible for inclusion.

Patients were excluded if they had conditions that influence bone metabolism or if they had taken bisphosphonates, parathyroid hormone (PTH), corticosteroids, or selective estrogen-receptor modulators. Patients were also excluded if they had active peptic ulcer, abnormal hepatic function, malignant disease, a history of severe brain stroke, or a history of parathyroidectomy.

BMD at the distal third of the radius was measured by using dual-energy X-ray absorptiometry on a DTX-200 densitometer before treatment and after 1 year. Dialysates with a calcium content of 2.5 mEq/l were used.

Biochemical parameters including phosphorus (P), calcium (Ca), whole PTH, total alkaline phosphatase (ALP), and albumin were measured by using standard laboratory techniques. Serum calcium values were corrected for serum albumin concentration by using the following formula: corrected calcium (mg/dl) = total calcium (mg/dl) + 4 − albumin (g/dl).

A combination of calcium-based phosphate binder, sevelamer, lanthanum carbonate hydrate, calcitriol, alfacalcidol, maxacalcitol, and cinacalcet was titrated according to the serum calcium, phosphate, or PTH levels.

Most of the laboratory tests were performed once a month and blood samples were withdrawn at the start of the second dialysis session of each week.

ALP level was measured as bone turnover marker [17].

Data were expressed as mean ± standard deviation (SD). Data comparisons between two groups were performed by* t*-test. Data for whole PTH were expressed as median and interquartile range. Data comparison for whole PTH levels was performed by using the Wilcoxon signed-rank test. *P* values of <0.05 were considered significant.

## 3. Results

Seventeen patients (mean age: 72.8 years) were treated with denosumab 60 mg every 6 months. Their original disease was diabetes mellitus in nine patients, hypertensive nephropathy in three patients, glomerulonephritis in three patients, and rapid progressive glomerulonephritis in one patient and it was unknown in one patient. One patient had a fragility fracture at baseline in the denosumab group. Twenty patients (mean age: 71.2 years) were not treated with denosumab (control group). Their original disease was diabetes mellitus in eleven patients, hypertensive nephropathy in two patients, and glomerulonephritis in five patients and it was unknown in two patients. Three patients in the control group had a fragility fracture at baseline.

None of the patients was excluded from the analysis. The baseline characteristics or parameters in both groups were not significantly different (Tables 1 and 2).

The administration of denosumab was clinically well tolerated. In the denosumab group, at 7 days, the mean serum albumin-corrected calcium (Ca [alb]) decreased from 9.2 ± 0.5 mg/dl to 8.5 ± 1.1 mg/dl (*P* < 0.01), and the mean serum P decreased from 5.0 ± 1.3 mg/dl to 4.2 ± 0.9 mg/dl (*P* < 0.01). At one month, serum albumin-corrected calcium (Ca [alb]) was 9.2 ± 0.9 mg/dl and mean serum P was 4.0 ± 1.1 mg/dl (Table 3). Five patients who showed hypocalcemia (<8.0 mg/dl at 1 week), without clinical symptom, recovered soon after increased doses of vitamin D receptor activators and/or calcium-based phosphate binder.

Tables 4(a) and 4(b) show mean medication doses and number of treated patients by each medication during the treatment course. Alfacalcidol appears to have been used more in the denosumab group, and maxacalcitol was used more in the control group.

ALP level decreased in the denosumab group at 1 year (Table 5) and showed decreased bone turnover.

At 1 year, BMD at the distal third of the radius increased by 2.6 ± 4.4% in the denosumab group and decreased by 4.5 ± 7.7% in the control group (*P* < 0.001) (Table 6).

## 4. Discussion

The present study demonstrated that male hemodialysis patients with low BMD who received subcutaneous administration of 60 mg of denosumab every 6 months had significantly increased BMD at the distal third of the radius at 12 months, in comparison with a control group.

Although the patient selection was not randomized, selection was not intentional, because denosumab was used as treatment only by one of the primary physicians. The primary physician was assigned randomly during the initial visit of each patient at the clinic.

BMD measurement alone is known to show no correlation with fracture risk in this population. However, as BMD is a major factor reflecting bone strength [18], it seems logical that low BMD alone would increase the risk of fractures, and monitoring BMD is therefore a sensible means of measuring therapeutic effects.

Denosumab therapy prevents the interaction of RANKL with RANK, its receptor, on osteoclasts and their precursors, thereby blocking the formation, function, and survival of osteoclasts. By contrast, bisphosphonates chemically bind to calcium hydroxyapatite in bone; they reduce bone resorption by blocking the function and survival but not the formation of osteoclasts.

Although successful studies concerning the use of denosumab in hemodialysis patients were reported, those studies did not have a control group [12–14]. Denosumab led to significant increase in lumbar spine BMD and femoral neck BMD but not radius BMD in eleven hemodialysis patients in one year [12]. We cannot explain why radius BMD increased in our study and did not significantly increase in the previous study. We speculate that if they compared the treated group with a control group, they may find a significant difference. The BMD increased in both the femoral neck (mean increase: 23.7%  ± 4.0%) and lumbar spine (17.1%  ± 2.6%) after 6 months in the hemodialysis patients with severe secondary hyperparathyroidism [13].

A meta-analysis of studies that reported on BMD and fractures in chronic kidney disease showed that BMD was significantly lower in the subjects with fractures than in those without fractures [5]. This study was too small to show the effect of therapy on the incidence of fracture.

The common adverse effect of denosumab is hypocalcemia. Two case reports warned against the use of denosumab because of severe hypocalcemia [15, 16]. In another case, hypocalcemia was also observed but was overcome with adjustment of the concomitant treatment [11]. Although we also observed hypocalcemia, we adjusted hypocalcemia by increasing calcium-based phosphate binder and/or vitamin D receptor activators. What is this mechanism? Secondary hyperparathyroidism is a hallmark of chronic renal failure. It results in accelerated bone resorption and bone formation. Calcium is supplied by bone resorption and utilized by bone formation. Denosumab reduces mainly bone resorption. This imbalance may cause hypocalcemia.

During the treatment course, four kinds of medications that increase Ca levels were used (Tables 4(a) and 4(b)). Those are calcium carbonate, alfacalcidol, calcitriol, and maxacalcitol. Cinacalcet reduced calcium levels. It appears that alfacalcidol was used more in the denosumab group, and maxacalcitol was used more in the control group. Which group was treated by stronger medications to increase Ca levels? A comparison was not possible, because no formula exists to convert the effects of each medication.

Our study has limitations. It is too small to evaluate the safety of denosumab and did not have a randomized control group. A large-scale, randomized controlled study is necessary to confirm the efficacy of the treatment. Bone biopsy with quantitative histomorphometric analysis is the gold standard for the diagnosis of renal osteodystrophy. Nonetheless, bone biopsy is an invasive procedure that is not routinely performed. We did not measure bone specific ALP, osteocalcin, TRACP-5b, and intact N-terminal propeptide of type I procollagen levels, which would have been informative for bone metabolism. We did not measure bone mineral density at the lumbar spine, total hip, or femoral neck.

In summary, in this observational study, denosumab administered at 6-month intervals over a period of 12 months increased bone mineral density at the distal third of the radius in male hemodialysis patients with low BMD. This result supports the continued investigation for the use of denosumab in the treatment for male hemodialysis patients with bone loss.

## Figures and Tables

**Table 1:** Baseline characteristics of the patients.

	Denosumab	Control	*P* value
*n*	17	20	
Age (years)	72.8 ± 9.5	71.2 ± 11.0	NS
Body mass index (kg/m^2^)	21.6 ± 2.3	20.8 ± 2.3	NS
HD duration (years)	7.1 ± 4.9	6.4 ± 5.3	NS
BMD (% of young mean)	56.7 ± 7.2	54.7 ± 11.0	NS

Continuous values are given as mean ± SD. The body mass index is the weight in kilograms divided by the square of the height in meters. BMD: bone mineral density; NS: not significant.

**Table 2:** Baseline parameters of the patients.

	Denosumab	Control	*P* value
Corrected Ca (mg/dl)	9.2 ± 0.5	9.0 ± 0.5	NS
Phosphate (mg/dl)	5.0 ± 1.3	4.8 ± 1.2	NS
W-PTH (pg/ml)	164 (58.5–228)	157 (108–231)	NS
ALP (U/l)	276 ± 129	270 ± 69	NS

W-PTH: whole PTH. Median and interquartile range are shown for whole PTH. The reference range for ALP level is from 115 to 359 U/l.

**Table 3:** Serum albumin-corrected calcium, P, and whole-PTH levels during the denosumab treatment course.

	Baseline*∗*	1 week*∗∗*	1 month	*P* value *∗* versus *∗∗*
Corrected Ca (mg/dl)	9.2 ± 0.5	8.5 ± 1.1	9.2 ± 0.9	*P* < 0.01
Phosphate (mg/dl)	5.0 ± 1.3	4.2 ± 0.9	4.0 ± 1.1	*P* < 0.01
W-PTH (pg/dl)	164 (58.5 to 228)	224 (96 to 355.5)	161 (82.5 to 234)	NS

W-PTH: whole PTH. Median and interquartile range are shown for whole PTH.

**Table:** (a) Mean medication dose in the treatment course of the denosumab group

	Baseline	3 months	6 months	12 months
CaCo_3_ (g/day)	1.47 (15)	1.65 (16)	1.18 (15)	1.32 (15)
Alfacalcidol (*μ*g/day)	0.31 (11)	0.43 (11)	0.34 (10)	0.34 (11)
Calcitriol (*μ*g/day)	0.01 (1)	0.03 (1)	0.03 (1)	0 (0)
Maxacalcitol (*μ*g/week)	5.0 (2)	4.41 (2)	2.94 (2)	4.71 (3)
Cinacalcet (mg/day)	2.94 (8)	2.94 (10)	2.94 (8)	4.41 (9)

The number in the parentheses denotes the number of patients treated with each medication among the 17 patients.

**Table:** (b) Mean medication dose in the treatment course in the control group

	Baseline	3 months	6 months	12 months
CaCo_3_ (g/day)	1.50 (14)	1.73 (15)	1.10 (15)	1.6 (15)
Alfacalcidol (*μ*g/day)	0.03 (2)	0.01 (1)	0.01 (1)	0.01 (1)
Calcitriol (*μ*g/day)	0 (0)	0 (0)	0 (0)	0 (0)
Maxacalcitol (*μ*g/week)	10.0 (7)	8.75 (7)	10.0 (6)	10.0 (14)
Cinacalcet (mg/day)	6.75 (16)	11.25 (17)	7.50 (17)	11.25 (17)

The number in the parentheses denotes the number of patients treated with each medication among the 20 patients.

**Table 5:** Parameters of the patients at 12 months.

	Denosumab group	Control group	*P* value
Corrected Ca (mg/dl)	9.1 ± 0.6	9.1 ± 0.4	NS
Phosphate (mg/dl)	5.1 ± 1.4	4.6 ± 0.9	NS
W-PTH (pg/ml)	128 (72 to 191)	140 (76 to 215)	NS
ALP (U/l)	185 ± 59	249 ± 65	*P* < 0.01

The reference range for ALP is from 115 to 359 U/l. Median and interquartile range are shown for whole PTH.

**Table 6:** BMD at the distal third of radius.

	Baseline (% of YAM)	1 year (% of YAM)	% change
Denosumab	56.7 ± 7.2	58 ± 7.9	2.6 ± 4.4%
Control	54.7 ± 11	52.3 ± 10.1	−4.5 ± 7.7%
*P* value			<0.001

BMD: bone mineral density; YAM: young adult mean.
